# Desmoid tumour in an inguinal hernia in a patient with a previous diagnosis of melanoma

**DOI:** 10.3332/ecancer.2022.1394

**Published:** 2022-05-19

**Authors:** Natalia Tissera, Yanina Pflüger, Federico Waisberg, Martín Ángel, Andrés Rodríguez, Tomas Soulé, Alejandro Pairola, Guido Lutter, Mora Amat, Diego Enrico, Matías Chacón

**Affiliations:** 1 Department of Oncology, Alexander Fleming Cancer Institute, Buenos Aires 1426, Argentina; 2 Department of Surgery, Alexander Fleming Cancer Institute, Buenos Aires 1426, Argentina; 3 Department of Pathology, Alexander Fleming Cancer Institute, Buenos Aires 1426, Argentina; a https://orcid.org/0000-0002-3396-6878; b https://orcid.org/0000-0003-4435-5068; c https://orcid.org/0000-0002-1463-8887; d https://orcid.org/0000-0002-0880-3153; e https://orcid.org/0000-0003-4121-6855; f https://orcid.org/0000-0001-6872-4185

**Keywords:** aggressive fibromatosis, inguinal hernia, melanoma, desmoid tumour, differential diagnosis

## Abstract

A 68-year-old man, without a family history of cancer, was treated for primary cutaneous melanoma of the scalp. Two years later, a right lateral cervical lymph recurrence was observed and he was treated with lymphadenectomy and adjuvant nivolumab for 1 year. Four years from the initial melanoma diagnosis, a computer tomography scan showed a solid nodular lesion of 26 × 40 × 75 mm inside the previously known inguinoscrotal hernia. A new recurrence of melanoma was the most probable diagnosis and a right inguinal hernioplasty was performed. Notably, the histopathological examination revealed a mesenteric fibromatosis with the typical immunohistochemical pattern (strong nuclear staining of β-catenin). Interestingly, this represents the first case of a patient with a mesenteric desmoid tumour presenting as an inguinal hernia masking a cutaneous melanoma recurrence.

## Introduction

Aggressive fibromatosis, also known as desmoid tumour (DT), is a benign, locally invasive, non-metastasising neoplasm with a very high rate of recurrence. This entity represents 0.03% of all neoplasms and less than 3% of all soft tissue tumours [1, 2].

DTs can arise in virtually any part of the body, and according to their main localisations, they are classified as extra-abdominal (60%), abdominal wall (25%) and intra-abdominal (8%–15%) [3, 4].

Mesenteric fibromatosis represent 0.73% of all abdominal tumours and accounts for up to 8% of all desmoid neoplasms [5]. Interestingly, the clinical presentation of a mesenteric DT as an inguinal hernia is a very rare phenomenon that has been scarcely reported.

Here, we report the first case of a patient, with prior diagnosis of melanoma, with a mesenteric DT presenting as an inguinal hernia. In this case report, Case Reports (CARE) guidelines were followed and the patient’s authorisation was obtained before manuscript submission.

## Case presentation

A 68-year-old man, without a family history of cancer, was diagnosed with a pT2N0M0 BRAF (v-raf murine sarcoma viral oncogene homolog B1) wild type primary cutaneous melanoma of the scalp in April 2017. The treatment approach included a wide tumour resection and a sentinel lymph node biopsy. After that, the proposed follow-up scheme was physical examination and computer tomography (CT) scans every 6 months. Of note, in the baseline CT scan, a small right inguinal hernia with adipose content was evidenced.

Two years after the initial diagnosis, an enlargement of a right lateral cervical lymph node was detected. A core needle biopsy confirmed the presence of melanoma recurrence. At this time, the patient underwent a right cervical lymph node dissection, obtaining 2 out of 23 lymph nodes with melanoma metastases. Adjuvant treatment with nivolumab was completed for 1 year with excellent tolerance.

**Figure 1.**
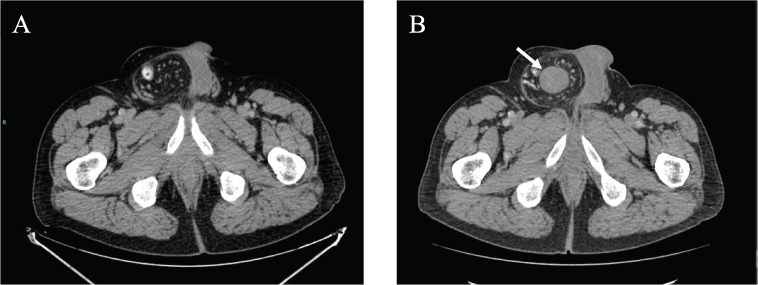
(a) September 2020 CT scan showing a right inguinal hernia. (b) September 2021 CT scan showing a solid nodular lesion of 26 × 40 × 75 mm inside the already-known inguinoscrotal hernia.

**Figure 2.**
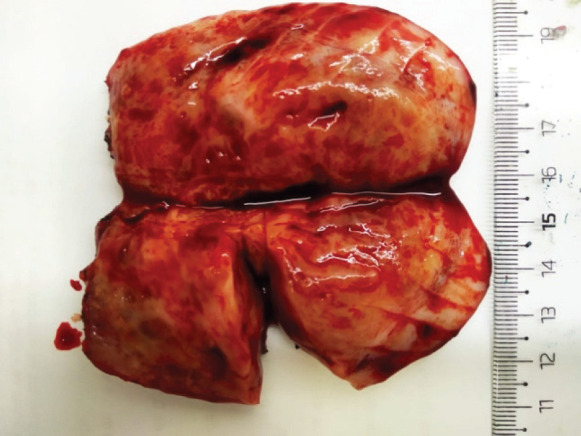
Macroscopic appearance of the solid inguinal mass.

**Figure 3.**
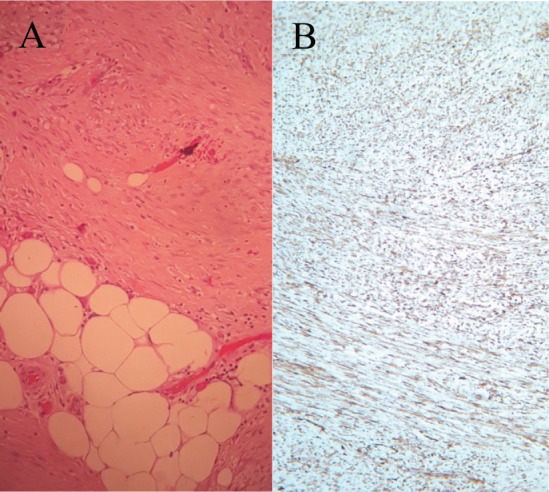
(a) Histological findings of a proliferation of tapered mesenchymal cells of oeosinophilic cytoplasm assembled between areas of collagen. (b) Strong nuclear staining of β-catenin was observed.

In September 2021, 4 years after the initial melanoma diagnosis, a follow-up CT scan showed a solid nodular lesion of 26 × 40 × 75 mm inside the already-known inguinoscrotal hernia (Figure 1). In this context, a diagnostic dilemma was raised. Presumptive diagnosis was a new recurrence of melanoma. In accordance with the tumour board decision, a right inguinal hernioplasty was performed and the solid nodular mass (70 × 30 × 30 mm) was removed (Figure 2). Histopathological examination revealed a proliferation of tapered mesenchymal cells of oeosinophilic cytoplasm assembled between areas of collagen. No mitotic figures or necrosis were observed. Immunohistochemical markers revealed that the tumour was negative for acute myeloid leukaemia (AML), anti-pan-cytokeratin AE1/AE3 (Cytokeratin AE1/AE3), cluster of differentiation 34 (CD34) and S100. Strong nuclear staining of β-catenin was observed (Figure 3). The final diagnosis was a mesenteric DT.

During the last follow-up exam (December 2021), the patient remained with no evidence of the disease.

## Discussion

DTs appear to be of aponeurotic origin, have a benign histological appearance and result from an aggressive fibroblast–myofibroblast proliferation [6]. John Macfarlane first described this disease more than 150 years ago [7, 8].

However, the real aetiology of mesenteric fibromatosis remains unknown. Most cases have been reported in association with Gardner’s syndrome, previous trauma and prolonged oestrogen exposure, but mesenteric fibromatosis can occur as a primary condition in the absence of any predisposing factors [9].

Atypical localisation of DT represents a diagnostic challenge. Watchful waiting is usually the first recommended approach.

This strategy is supported by the occurrence of spontaneous regression. In a recent presentation, Bonvalot *et al* [10] reported an incidence of 20% of tumour regression in a retrospective cohort of 147 patients. In addition, a phase II observational trial included 100 patients who were proposed active surveillance as the therapeutic approach. With 46.6 months of follow-up, less than 10% of the patients required radiotherapy or surgery. The authors concluded that active surveillance of newly diagnosed DT is an effective strategy, sparing patients from unnecessary invasive treatments [11].

The current guidelines also highlight the importance of offering active surveillance in patients with newly diagnosed DTs [12]. A rapid diagnosis of DT is essential to offer non-operative management for these tumours.

In progressive DTs, other therapeutic recommendations include anti-oestrogens, non-steroidal anti-inflammatory drugs, tyrosine kinase inhibitors and chemotherapy. Local treatments are often indicated in tumours with life-threatening localisations, such as lesions arising in the root of the mesentery [13].

In this context, if a DT is clinically or radiologically suspected, a biopsy with a 14–16G Tru-Cut needle is recommended and the samples must be reviewed by an expert pathologist in soft tissue tumours, otherwise up to 30%–40% of the cases may be incorrectly diagnosed [14]. In our particular case, given the mesenteric localisation and the patient’s symptoms, tumour resection would also have been probably offered if a prior diagnosis had been established. Nonetheless, it should be considered that early suspicion of DT may contribute to the avoidance of unnecessary surgical excisions.

Taking this point into consideration, we carried out a literature review in PubMed and Scopus databases, including articles describing DT presenting as inguinal masses. Abstract selection and data extraction were made by two investigators separately.

Since 1999, only nine cases of DT that were diagnosed in the context of an inguinal hernia were reported. As in our case, this atypical presentation led to unusual treatment strategies for DTs. Table 1 summarises the main case characteristics of selected studies.

The median age was 30 years (range: 14–70 years) and the inguinal hernia was right-sided in five cases (56%) and left-sided in three reports (33%). All of the included cases were men. In only one tumour, immunohistochemical β-catenin staining was performed, which confirmed the diagnosis of a mesenteric DT. Tumour resection was performed in all of the case reports. Further resection of other structures, such as the ileum or the omentum, was required in three patients. It should be highlighted that in two patients an orchiectomy was performed. No considerations regarding fertility preservation, adenomatous polyposis coli (APC) gene status or history of familial adenomatous polyposis (FAP) were reported in the selected reports.

Clinical reasoning to support treatment decisions was not evident in the studies analysed as none of the included reports considered or suspected a diagnosis of DT before the initial surgical approach. Additionally, in most of the evaluated reports, patients referred mild or vague symptoms.

In the analysed studies, the follow-up was short or not reported. Resultantly, no conclusions regarding the efficacy of a surgical approach for this atypical presentation can be obtained. In our case, there were no clinical or radiological findings to suspect DT. The finding of a painful inguinal mass in a patient with a prior diagnosis of relapsed melanoma led the tumour board to recommend surgical resection and the concomitant inguinal hernioplasty.

It may be argued that for most soft tissue sarcomas, a core needle biopsy is the preferred diagnostic method unless the clinical scenario supports a more aggressive strategy. Consequently, the discussion within a multidisciplinary tumour board is essential to define possible differential diagnoses and the best diagnostic and therapeutic procedures.

None of the reported cases had a previous diagnosis or suspicion of DT to consider other treatment strategies such as watchful waiting approaches or systemic therapies in case of life-threatening complications, instead of upfront surgery.

Another lesson that might be taken from our case is not to associate every new tumoural finding with a patient’s prior cancer history. However, to the best of our knowledge, there is no other report of DT in a patient with a prior melanoma diagnosis. Considering that the patient had no familial history of cancer, there is a low probability of a germline association between these two entities.

## Conclusion

A comprehensive and better understanding of DT may help to treat these tumours with a more accurate approach regardless of the atypical sites of presentation. As observed in our case and the available literature reports, DT was not proposed as a differential diagnosis of a mass localised in an inguinal hernia. In most scenarios, and especially in subjects with medical comorbidities, early recognition of this entity may prove essential to avoiding surgical resections and preserving patients’ quality of life. Considering that the patient had no familial history of cancer, and with the current knowledge, there is a low probability of a germline association between these entities.

## Conflicts of interest

The authors declare that there are no conflicts of interest.

## Funding

None.

## Appendix

The following query was used for the literature review:

“fibromatosis, abdominal”[MeSH Terms] OR (“fibromatosis”[All Fields] AND “abdominal”[All Fields]) OR “abdominal fibromatosis”[All Fields] OR (“mesenteric”[All Fields] AND “fibromatosis”[All Fields]) OR “mesenteric fibromatosis”[All Fields] OR (“fibromatosis, aggressive”[MeSH Terms] OR (“fibromatosis”[All Fields] AND “aggressive”[All Fields]) OR “aggressive fibromatosis”[All Fields] OR (“desmoid”[All Fields] AND “tumor”[All Fields]) OR “desmoid tumor”[All Fields]) OR ((“fibromatosis, aggressive”[MeSH Terms] OR (“fibromatosis”[All Fields] AND “aggressive”[All Fields]) OR “aggressive fibromatosis”[All Fields] OR (“aggressive”[All Fields] AND “fibromatosis”[All Fields])) AND (“hernia, inguinal”[MeSH Terms] OR (“hernia”[All Fields] AND “inguinal”[All Fields]) OR “inguinal hernia”[All Fields] OR (“inguinal”[All Fields] AND “hernia”[All Fields]))).

## Figures and Tables

**Table 1.** Review of previously reported cases of DT in an inguinal hernia.

Reference	Inguinal hernia localisation	Age, Sex	Symptoms	Treatment	Follow-up without recurrence (months)
Lam *et al* [15]	Left	37, M	Irreducible hernia, asymptomatic	Tumour and omentum resection. Herniorrhaphy, and orchidectomy	4
Hokuto *et al* [16]	Right	70, M	Inguinal swelling	Herniorrhaphy and tumour resection	NR
Alsaif *et al* [17]	Left	52, M	Inguinal swelling and growing mass	Tumour and ileal segment resection. Omentectomy	NR
Groh *et al* [18]	Right	24, M	Scrotal swelling	Tumour resection and hernioplasty	NR
Heyse *et al* [19]	NR	24, M	Bowel obstruction	Hernioplasty	NR
Pradhan *et al* [20]	Right	14, M	Inguinal swelling and growing mass	Wide local excision	4.5
Tian *et al* [21]	Right	26, M	Irreducible hernia, asymptomatic	Tumour resection and omentectomy	NR
Khoo *et al* [22]	Right	51, M	Scrotal swelling and growing mass	Tumour resection and right orchiectomy	NR
Liu *et al* [23]	Left	30, M	Epididymal mass and scrotal vague discomfort	Hernioplasty. Tumour and omentum resection.	1.5

M, male; NR, no reported.

## References

[1] Reitamo JJ, Häyry P, Nykyri E (1982). The desmoid tumor. I. Incidence, sex-, age- and anatomical distribution in the Finnish population. Am J Clin Pathol.

[2] Bn A, Cd JK, Ps S (2015). Giant aggressive mesenteric fibromatosis- a case report. J Clin Diagn Res JCDR.

[3] Nieuwenhuis MH, Casparie M, Mathus-Vliegen LMH (2011). A nation-wide study comparing sporadic and familial adenomatous polyposis-related desmoid-type fibromatoses. Int J Cancer.

[4] Li Destri G, Ferraro MJ, Calabrini M (2014). Desmoid-type fibromatosis of the mesentery: report of a sporadic case with emphasis on differential diagnostic problems. Case Rep Med.

[5] Abate M, Pigazzi A (2018). Mesenteric fibromatosis in a patient with a history of neuroblastoma: a case report. J Surg Case Rep.

[6] Nicolas G, Kfoury T, Shimlati R (2016). Incidental finding and management of mesenteric fibromatosis. Am J Case Rep.

[7] Sinukumar S, Gomes RM, Kumar RK (2014). Sporadic giant mesenteric fibromatosis. Indian J Surg Oncol.

[8] Otero S, Moskovic EC, Strauss DC (2015). Desmoid-type fibromatosis. Clin Radiol.

[9] Gari MKM, Guraya SY, Hussein AM (2012). Giant mesenteric fibromatosis: report of a case and review of the literature. World J Gastrointest Surg.

[10] Bonvalot S, TernTernSuand Fiore M (2013). Spontaneous regression of primary abdominal wall desmoid tumors: more common than previously thought. Ann Surg Oncol.

[11] Bonvalot S, Lam L, Le Cesne A (2021). 1523MO Initial active surveillance strategy for patients with peripheral sporadic desmoids: a multicentre phase II observational trial. Ann Oncol.

[12] Alman B, Attia S, Baumgarten C (2020). The management of desmoid tumours: a joint global consensus-based guideline approach for adult and paediatric patients. Eur J Cancer.

[13] Spear MA, Jennings LC, Mankin HJ (1998). Individualizing management of aggressive fibromatoses. Int J Radiat Oncol Biol Phys.

[14] Kasper B, Baumgarten C, Garcia J (2017). An update on the management of sporadic desmoid-type fibromatosis: a European Consensus Initiative between Sarcoma PAtients EuroNet (SPAEN) and European Organization for Research and Treatment of Cancer (EORTC)/Soft Tissue and Bone Sarcoma Group (STBSG). Ann Oncol.

[15] Lam K, Lo C, Lee M (1999). Omental fibromatosis presenting as an incarcerated inguinal hernia. Aust N Z J Surg.

[16] Hokuto D, Okayama J, Kuge H (2006). A case of an omental desmoid tumor discovered as the content of an inguinal hernia. Nihon Shokaki Geka Gakkai Zasshi.

[17] Alsaif FA (2011). Mesenteric fibromatosis presenting as an irreducible inguinal hernia. Saudi J Gastroenterol Off J Saudi Gastroenterol Assoc.

[18] Groh OR, Kouwenberg L, Southwold A (2012). Een man met een scrotale zwelling. Ned Tijdschr Geneeskd.

[19] Heyse P, Moore A, Hendershot K (2013). Mesenteric fibromatosis: case report. Eur J Surg Sci.

[20] Pradhan A, Tudu D, Nayak M (2015). Desmoid tumor in a scar from inguinal hernia repair: a case report. ISOR J.

[21] Tian C, Yip J, Lo OSH (2017). Primary omental fibromatosis presenting as an incarcerated inguinal hernia: case report from a single institution over 20 years. Surg Pract.

[22] Khoo PJ, Jacob S (2017). An omental fibroma resembling a testicular tumour but presented as an irreducible inguinal hernia. J Surg Case Rep.

[23] Liu JY, Li SQ, Yao SJ (2021). Omental mass combined with indirect inguinal hernia leads to a scrotal mass: a case report. World J Clin Cases.

